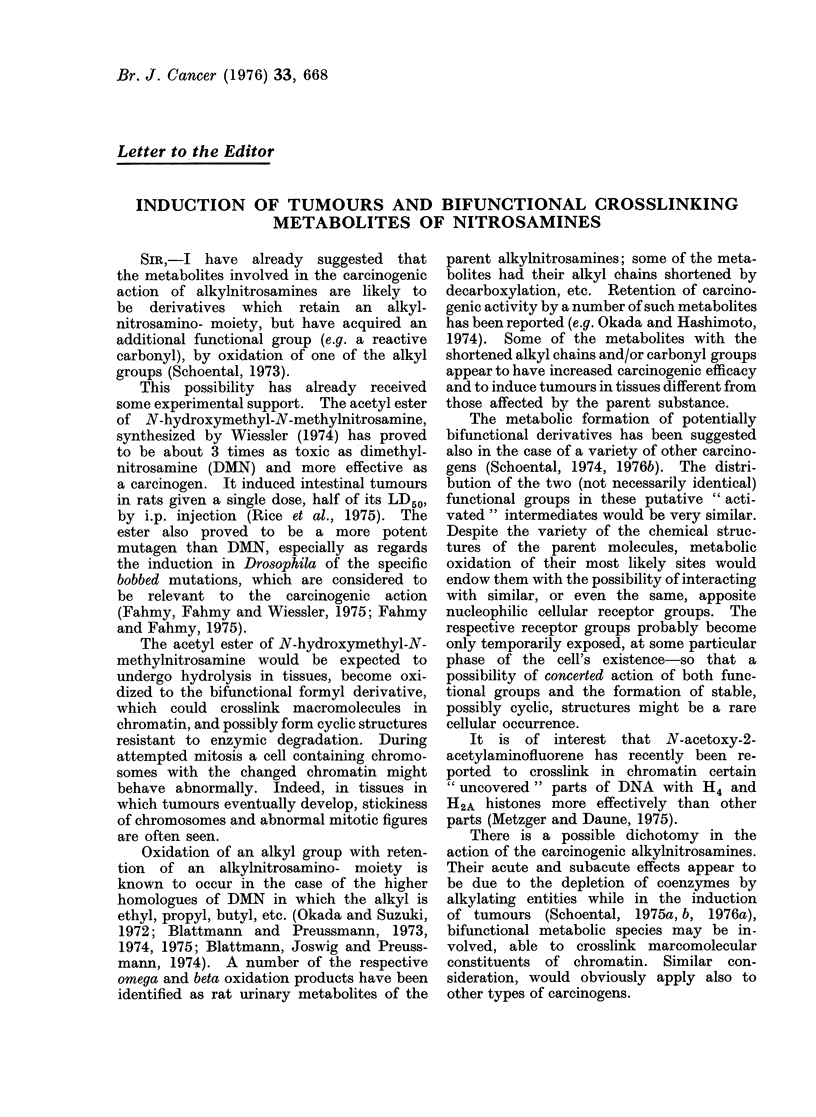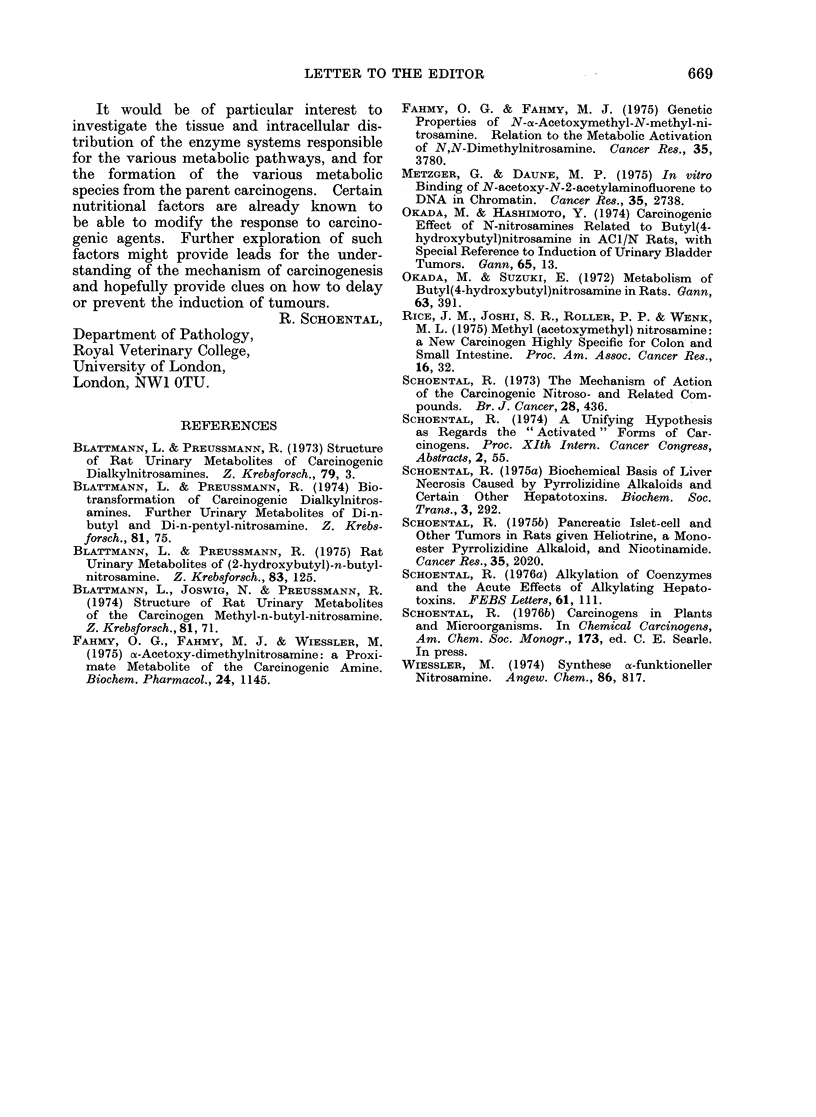# Letter: Induction of tumours and bifunctional crosslinking metabolites of nitrosamines.

**DOI:** 10.1038/bjc.1976.106

**Published:** 1976-06

**Authors:** R. Schoental


Br. J. Cancer (1976) 33, 668

### Letter to the Editor

### INDUCTION OF TUMOURS AND BIFUNCTIONAL CROSSLINKING METABOLITES OF NITROSAMINES

SIR,—I have already suggested that the metabolites involved in the carcinogenic action of alkylnitrosamines are likely to be derivatives which retain an alkylnitrosamino- moiety, but have acquired an additional functional group (*e.g.* a reactive carbonyl), by oxidation of one of the alkyl groups (Schoental, 1973).

This possibility has already received some experimental support. The acetyl ester of *N*-hydroxymethyl-*N*-methylnitrosamine, synthesized by Wiessler (1974) has proved to be about 3 times as toxic as dimethylnitrosamine (DMN) and more effective as a carcinogen. It induced intestinal tumours in rats given a single dose, half of its $LD_{50}$, by i.p. injection (Rice *et al.*, 1975). The ester also proved to be a more potent mutagen than DMN, especially as regards the induction in *Drosophila* of the specific *bobbed* mutations, which are considered to be relevant to the carcinogenic action (Fahmy, Fahmy and Wiessler, 1975; Fahmy and Fahmy, 1975).

The acetyl ester of *N*-hydroxymethyl-*N*-methylnitrosamine would be expected to undergo hydrolysis in tissues, become oxidized to the bifunctional formyl derivative, which could crosslink macromolecules in chromatin, and possibly form cyclic structures resistant to enzymic degradation. During attempted mitosis a cell containing chromosomes with the changed chromatin might behave abnormally. Indeed, in tissues in which tumours eventually develop, stickiness of chromosomes and abnormal mitotic figures are often seen.

Oxidation of an alkyl group with retention of an alkylnitrosamino- moiety is known to occur in the case of the higher homologues of DMN in which the alkyl is ethyl, propyl, butyl, etc. (Okada and Suzuki, 1972; Blattmann and Preussmann, 1973, 1974, 1975; Blattmann, Joswig and Preussmann, 1974). A number of the respective *omega* and *beta* oxidation products have been identified as rat urinary metabolites of the parent alkylnitrosamines; some of the metabolites had their alkyl chains shortened by decarboxylation, etc. Retention of carcinogenic activity by a number of such metabolites has been reported (*e.g.* Okada and Hashimoto, 1974). Some of the metabolites with the shortened alkyl chains and/or carbonyl groups appear to have increased carcinogenic efficacy and to induce tumours in tissues different from those affected by the parent substance.

The metabolic formation of potentially bifunctional derivatives has been suggested also in the case of a variety of other carcinogens (Schoental, 1974, 1976*b*). The distribution of the two (not necessarily identical) functional groups in these putative "activated" intermediates would be very similar. Despite the variety of the chemical structures of the parent molecules, metabolic oxidation of their most likely sites would endow them with the possibility of interacting with similar, or even the same, apposite nucleophilic cellular receptor groups. The respective receptor groups probably become only temporarily exposed, at some particular phase of the cell's existence—so that a possibility of *concerted* action of both functional groups and the formation of stable, possibly cyclic, structures might be a rare cellular occurrence.

It is of interest that *N*-acetoxy-2-acetylaminofluorene has recently been reported to crosslink in chromatin certain "uncovered" parts of DNA with $H_4$ and $H_{2A}$ histones more effectively than other parts (Metzger and Daune, 1975).

There is a possible dichotomy in the action of the carcinogenic alkylnitrosamines. Their acute and subacute effects appear to be due to the depletion of coenzymes by alkylating entities while in the induction of tumours (Schoental, 1975*a*, *b*, 1976*a*), bifunctional metabolic species may be involved, able to crosslink marcomolecular constituents of chromatin. Similar consideration, would obviously apply also to other types of carcinogens.

It would be of particular interest to investigate the tissue and intracellular distribution of the enzyme systems responsible for the various metabolic pathways, and for the formation of the various metabolic species from the parent carcinogens. Certain nutritional factors are already known to be able to modify the response to carcinogenic agents. Further exploration of such factors might provide leads for the understanding of the mechanism of carcinogenesis and hopefully provide clues on how to delay or prevent the induction of tumours.

R. SCHOENTAL,

Department of Pathology,
Royal Veterinary College,
University of London,
London, NW1 0TU.

REFERENCES

BLATTMANN, L. & PREUSSMANN, R. (1973) Structure of Rat Urinary Metabolites of Carcinogenic Dialkylnitrosamines. *Z. Krebsforsch.*, **79**, 3.

BLATTMANN, L. & PREUSSMANN, R. (1974) Biotransformation of Carcinogenic Dialkylnitrosamines. Further Urinary Metabolites of Di-n-butyl and Di-n-pentyl-nitrosamine. *Z. Krebsforsch.*, **81**, 75.

BLATTMANN, L. & PREUSSMANN, R. (1975) Rat Urinary Metabolites of (2-hydroxybutyl)-*n*-butylnitrosamine. *Z. Krebsforsch.*, **83**, 125.

BLATTMANN, L., JOSWIG, N. & PREUSSMANN, R. (1974) Structure of Rat Urinary Metabolites of the Carcinogen Methyl-n-butyl-nitrosamine. *Z. Krebsforsch.*, **81**, 71.

FAHMY, O. G., FAHMY, M. J. & WIESSLER, M. (1975) α-Acetoxy-dimethylnitrosamine: a Proximate Metabolite of the Carcinogenic Amine. *Biochem. Pharmacol.*, **24**, 1145.

FAHMY, O. G. & FAHMY, M. J. (1975) Genetic Properties of *N*-α-Acetoxymethyl-*N*-methyl-nitrosamine. Relation to the Metabolic Activation of *N*,*N*-Dimethylnitrosamine. *Cancer Res.*, **35**, 3780.

METZGER, G. & DAUNE, M. P. (1975) *In vitro* Binding of *N*-acetoxy-*N*-2-acetylaminofluorene to DNA in Chromatin. *Cancer Res.*, **35**, 2738.

OKADA, M. & HASHIMOTO, Y. (1974) Carcinogenic Effect of N-nitrosamines Related to Butyl(4-hydroxybutyl)nitrosamine in ACl/N Rats, with Special Reference to Induction of Urinary Bladder Tumors. *Gann*, **65**, 13.

OKADA, M. & SUZUKI, E. (1972) Metabolism of Butyl(4-hydroxybutyl)nitrosamine in Rats. *Gann*, **63**, 391.

RICE, J. M., JOSHI, S. R., ROLLER, P. P. & WENK, M. L. (1975) Methyl (acetoxymethyl) nitrosamine: a New Carcinogen Highly Specific for Colon and Small Intestine. *Proc. Am. Assoc. Cancer Res.*, **16**, 32.

SCHOENTAL, R. (1973) The Mechanism of Action of the Carcinogenic Nitroso- and Related Compounds. *Br. J. Cancer*, **28**, 436.

SCHOENTAL, R. (1974) A Unifying Hypothesis as Regards the "Activated" Forms of Carcinogens. *Proc. XIth Intern. Cancer Congress, Abstracts*, **2**, 55.

SCHOENTAL, R. (1975*a*) Biochemical Basis of Liver Necrosis Caused by Pyrrolizidine Alkaloids and Certain Other Hepatotoxins. *Biochem. Soc. Trans.*, **3**, 292.

SCHOENTAL, R. (1975*b*) Pancreatic Islet-cell and Other Tumors in Rats given Heliotrine, a Monoester Pyrrolizidine Alkaloid, and Nicotinamide. *Cancer Res.*, **35**, 2020.

SCHOENTAL, R. (1976*a*) Alkylation of Coenzymes and the Acute Effects of Alkylating Hepatotoxins. *FEBS Letters*, **61**, 111.

SCHOENTAL, R. (1976*b*) Carcinogens in Plants and Microorganisms. In *Chemical Carcinogens, Am. Chem. Soc. Monogr.*, **173**, ed. C. E. Searle. In press.

WIESSLER, M. (1974) Synthese α-funktioneller Nitrosamine. *Angew. Chem.*, **86**, 817.